# Supplementary material for: A UMLS-based spell checker for natural language processing in vaccine safety
Source: BMC Med Inform Decis Mak. 2007 Feb 12;7:3. doi: 10.1186/1472-6947-7-3 (PMC1805499; doi:10.1186/1472-6947-7-3)
Supplement: Additional file 13 — RAP application directory. Sets up directory for RAP (RDF application for PHP) source code [file 1472-6947-7-3-S13.gz › rap/doc/phpdoc/default/VCARD.html]

xml version="1.0" encoding="iso-8859-1"?


Docs For Class VCARD


## Class VCARD

Description

Description |
Methods (details)

Located in /vocabulary/VCARD\_C.php (line 21)

Method Summary

Description |
Methods (details)

void
ADR
()

void
ADRPROPERTIES
()

void
ADRTYPES
()

void
AGENT
()

void
BDAY
()

void
CATEGORIES
()

void
COUNTRY
()

void
EMAIL
()

void
EMAILTYPES
()

void
EXTADD
()

void
FAMILY
()

void
FN
()

void
GEO
()

void
GIVEN
()

void
GROUP
()

void
KEY
()

void
LABEL
()

void
LOCALITY
()

void
MAILER
()

void
N
()

void
NAME
()

void
NICKNAME
()

void
NPROPERTIES
()

void
ORGNAME
()

void
ORGPROPERTIES
()

void
ORGUNIT
()

void
OTHER
()

void
PCODE
()

void
PHOTO
()

void
POBOX
()

void
PREFIX
()

void
PRODID
()

void
REGION
()

void
REV
()

void
ROLE
()

void
SORT\_STRING
()

void
SOUND
()

void
SOURCE
()

void
STREET
()

void
SUFFIX
()

void
TEL
()

void
TELTYPES
()

void
TITLE
()

void
TZ
()

void
TZTYPES
()

void
UID
()

void
VCARD\_CLASS
()

Methods

Description |
Methods (details)

ADR (line 162)

void

ADR
()

ADRPROPERTIES (line 60)

void

ADRPROPERTIES
()

ADRTYPES (line 36)

void

ADRTYPES
()

AGENT (line 78)

void

AGENT
()

BDAY (line 90)

void

BDAY
()

CATEGORIES (line 114)

void

CATEGORIES
()

COUNTRY (line 222)

void

COUNTRY
()

EMAIL (line 192)

void

EMAIL
()

EMAILTYPES (line 48)

void

EMAILTYPES
()

EXTADD (line 180)

void

EXTADD
()

FAMILY (line 198)

void

FAMILY
()

FN (line 144)

void

FN
()

GEO (line 174)

void

GEO
()

GIVEN (line 276)

void

GIVEN
()

GROUP (line 186)

void

GROUP
()

KEY (line 264)

void

KEY
()

LABEL (line 282)

void

LABEL
()

LOCALITY (line 252)

void

LOCALITY
()

MAILER (line 240)

void

MAILER
()

N (line 120)

void

N
()

NAME (line 210)

void

NAME
()

NICKNAME (line 294)

void

NICKNAME
()

NPROPERTIES (line 42)

void

NPROPERTIES
()

ORGNAME (line 108)

void

ORGNAME
()

ORGPROPERTIES (line 30)

void

ORGPROPERTIES
()

ORGUNIT (line 216)

void

ORGUNIT
()

OTHER (line 246)

void

OTHER
()

PCODE (line 126)

void

PCODE
()

PHOTO (line 138)

void

PHOTO
()

POBOX (line 258)

void

POBOX
()

PREFIX (line 132)

void

PREFIX
()

PRODID (line 270)

void

PRODID
()

REGION (line 168)

void

REGION
()

REV (line 96)

void

REV
()

ROLE (line 300)

void

ROLE
()

SORT\_STRING (line 102)

void

SORT\_STRING
()

SOUND (line 228)

void

SOUND
()

SOURCE (line 84)

void

SOURCE
()

STREET (line 72)

void

STREET
()

SUFFIX (line 150)

void

SUFFIX
()

TEL (line 288)

void

TEL
()

TELTYPES (line 54)

void

TELTYPES
()

TITLE (line 234)

void

TITLE
()

TZ (line 204)

void

TZ
()

TZTYPES (line 66)

void

TZTYPES
()

UID (line 24)

void

UID
()

VCARD\_CLASS (line 156)

void

VCARD\_CLASS
()

Documentation generated on Fri, 17 Dec 2004 16:18:17 +0100 by phpDocumentor 1.3.0RC3
